# Nanosponges by the oxo-Michael polyaddition of cyclodextrins as sorbents of water pollutants: the *o*-toluidine case

**DOI:** 10.1007/s11356-022-22501-2

**Published:** 2022-08-24

**Authors:** Valentina Pifferi, Elena Ferrari, Amedea Manfredi, Paolo Ferruti, Jenny Alongi, Elisabetta Ranucci, Luigi Falciola

**Affiliations:** Dipartimento Di Chimica, Università Degli Studi Di Milano, via C. Golgi 19, 20133 Milano, Italy

**Keywords:** Cyclodextrin nanosponges, 1,4-Bisacryloylpiperazine, 2,2-Bisacrylamidoacetic acid, *o*-Toluidine sorption in water, Voltammetric detection

## Abstract

Hydrophilic cyclodextrin nanosponges were prepared by the oxo-Michael polyaddition in an aqueous solution at pH > 10 of α-, β-, and γ-cyclodextrin with 1,4-bisacryloylpiperazine or 2,2-bisacrylamidoacetic acid. These nanosponges and, for comparison purposes, their precursor cyclodextrins were tested as sorbents of *o*-toluidine, a carcinogenic wastewater contaminant, by monitoring the depletion of *o*-toluidine from a 10^−4^ M (10 ppm) aqueous solutions. To this aim, an innovative analytical procedure was used: The voltammetric peak currents of *o*-toluidine in linear sweep voltammetry experiments were registered using multi-walled carbon nanotubes-modified glassy carbon electrodes. The experimental sorption curves fitted a mono-exponential kinetic model, and the residual *o*-toluidine was 0.16 ppm, one order of magnitude lower than those of all other sorbents reported so far. The sorption capacities ranged from 88 to 199 µmol g^−1^ (10–21.3 mg g^−1^), equal to or higher than those of the parent cyclodextrins. All nanosponges were completely regenerated by extracting with methanol. After regeneration, the sorption capacity slightly improved, suggesting a rearrangement of the nanosponge network. Overall, it may be reasonably concluded that the cyclodextrin nanosponges reported in this paper warrant potential as *o*-toluidine exhaustive sorbents.

## Introduction

*o*-Toluidine is an important organic intermediate used, *inter alia*, in the production of dyes, synthetic rubber, chemicals, and, as a curing agent, epoxy resins. In addition, it is a major component of tobacco smoke (OECD 2004) and, consequently, ubiquitous in the human environment. *o*-Toluidine manufacturing and processing have produced increasing amounts of toluidine-polluted wastewaters, eventually merging into water bodies. *o*-Toluidine is classified by the International Agency for Research on Cancer (IARC) as “carcinogenic to humans” (group 1) (Baan et al. 2008); therefore, its efficient removal from water is required. Different technologies are currently available to remove *o*-toluidine from wastewater, including photodegradation (Cappelletti et al. 2015; Pifferi et al. 2015; San and Çjnar 2016), electrochemical treatment (López-Grimau et al. 2013), oxidation (Anotai et al. 2011), microbial digestion (Liu et al. 2002), and adsorption. Among them, adsorption is reckoned as one of the most attractive and effective purification techniques in wastewater treatment. Various sorbents have been reported in the literature (Elgarahy et al. 2021; El-Sayed et al. 2019) and in particular for anilines, including *o*-toluidine, such as organo-clay (Bishop et al. 2002; Zhang and Sparks 1993), montmorillonite (Essington 1994), silica (Voumard et al. 1995), rubber tire (Gupta et al. 2012), zeolites (Titus et al. 2002), activated carbon in powder, granular, or fiber form (Duman and Ayranci 2005) and polymers (Jianguo et al. 2005).

Cyclodextrins (CDs) are cyclic oligosaccharides formed by 6 (α-CD), 7 (β-CD), or 8 (γ-CD) glucopyranose units. They are well known to possess a hydrophobic cavity forming inclusion complexes with organic molecules through host-guest interactions, with dimensional selectivity due to the different sizes of their inner cavity (Dodziuk 2006; Szejtli 1998). CD-containing polymers, either linear (Janus et al. 1999) or immobilized on solid supports (Allabashi et al. 2007; Fan et al. 2003; Faraji et al. 2011) or crosslinked CD resins (Crini et al. 1999; García-Zubiri et al. 2007; Romo et al. 2008; Wilson et al. 2011; Yamasaki et al. 2006; Yu et al. 2003) were extensively studied as sorbents of organic pollutants with removal efficacy ranging within ample limits (Crini 2005, Yadav et al. 2022). Since the late nineties, hyper-crosslinked CDs have commonly been referred to as nanosponges (Ahmed et al. 2013; Li and Ma 1999). Nanosponges are supramolecular cage-like architectures in which the CD units are connected by many short moieties forming nanochannels (Bilensoy 2011; Krabicov et al. 2020; Mhlanga et al. 2007; Petitjean et al. 2021; Rizzi et al. 2021; Sherje et al. 2017; Taka et al. 2017; Trotta et al. 2012; Varana et al. 2020). Nanosponges are nanoporous by definition and show high inclusion constants with many organic pollutants, including aromatic and chlorinated compounds (Berto et al. 2007a and 2007b; Gupta et al. 2012; Sun et al. 2010; Rizzi et al. 2021; Yadav et al. 2022). Most nanosponges are based on β-CD, the cheapest CD on the market, and only a few examples of α- and γ-CD nanosponges have been reported (Cavalli et al. 2010). Nanosponges were obtained by interconnecting the CD molecules with different crosslinking agents, such as N,N’-carbonyldiimidazole (Ansari et al. 2011), triphosgene (Trotta 2011), diphenyl carbonate (Swaminathan et al. 2013), or organic dianhydrides (Mognetti et al. 2021). For all of them, the coupling reactions demanded organic solvents. Subsequently, β-CD-based nanosponges were synthesized in water at pH > 12 by the Michael oxo-polyaddition of β-CD, or β-CD/2-methylpiperazine mixtures, with 2,2-bisacrylamidoacetic acid (Swaminathan et al. 2010). Under these conditions, β -CD acted as a multifunctional monomer and gave rise to a previously undescribed type of nanosponges. In the present work, this preparation method was extended to α- and γ-CD, and a small library of CD nanosponges was prepared by polyaddition of all three CD’s with either 1,4-bisacryloylpiperazine or 2,2-bisacrylamidoacetic acid. These nanosponges were then tested as sorbents to remove *o*-toluidine from water and compared in this regard with their CD precursors. *o*-Toluidine sorption was monitored by using a previously optimized electroanalytical method based on linear sweep voltammetry (LSV) at multi-walled carbon nanotubes-modified glassy carbon electrode (Mardegan et al. 2014; Pifferi et al. 2014). The accuracy, sensitivity, and robustness of this optimized method were already demonstrated in previous papers on the degradation of *o*-toluidine (Cappelletti et al. 2015; Pifferi et al. 2015).

## Experimental

### Materials

Solvents and reagents, unless otherwise indicated, were analytical-grade commercial products and used as received considering the grade declared by the dealer. α-Cyclodextrin (α-CD) (89.5%) was purchased from Alfa Aesar, β-cyclodextrin (β-CD) (95%) from Fluka and γ-cyclodextrin (γ-CD) (97%) from ABCR. Lithium hydroxide monohydrate (LiOH•H_2_O) (99%), ethanol (>99.8%), and methanol (99%) were purchased from Sigma Aldrich. 2,2-Bisacrylamidoacetic acid (BAC) and 1,4-bisacryloylpiperazine (BP) were prepared as previously reported (Ferruti et al. 1999; Ferruti 1985). Distilled water (18 MΩ cm) purified with a Millipore Milli-Q apparatus was used in all experiments.

### Synthesis of CD-bisacrylamide nanosponges

#### Synthesis of BAC-based CD nanosponges

In a typical procedure, a solution of α-CD (3.125 g, 2.9 mmol) and LiOH•H_2_O (0.357 g, 8.4 mmol) in water (1.9 mL) was mixed with an aqueous solution of BAC (2.135 g, 10.6 mmol) and LiOH•H_2_O (0.445 g, 10.6 mmol) in 2.2 mL water. The mixture was allowed to react for 24 h at 25 ℃. The final product appeared as a homogeneous, transparent, and soft nanosponge, which was first soaked for 2 h in 50 mL deionized water, allowed for swelling for a further 2 h, brought to pH 5 with 37% HCl, and soaked for 2 h in ethanol (50 mL). This extracting procedure was repeated three times. The extracted product was finally dried to constant weight. Yield: 5.26 g (69%).

BAC-β-CD and BAC-γ-CD were prepared similarly. The reagents used in the preparation of all BAC nanosponges and their amounts were as follows.

Synthesis of BAC-β-CD nanosponges: β-CD (3.674 g, 3.1 mmol), LiOH•H_2_O (0.389 g, 9.2 mmol), H_2_O (2.4 mL), BAC (1.887 g, 9.4 mmol), LiOH•H_2_O (0.396 g, 9.4 mmol), H_2_O (1.8 mL). Yield: 76.2% (3.76 g).

Synthesis of BAC-γ-CD nanosponges: γ-CD (3.607 g, 2.7 mmol), LiOH•H_2_O (0.554 mg, 13.1 mmol), H_2_O (2.7 mL), BAC (1.347 g, 6.7 mmol), LiOH•H_2_O (0.285 mg, 6.7 mmol), H_2_O (1.2 mL). Yield: 47.4% (4.96 g).

#### Synthesis of BP-based CD nanosponges

BP-α-CD and BP-β-CD were prepared following the same procedure described for BAC-α-CD by substituting BP for equimolar amounts of BAC, apart from the fact that BP was dissolved in plain water before mixing with the CD/LiOH solution. The same reaction performed with γ-CD failed to produce insoluble crosslinked nanosponges. In detail, the reagents and amounts used were as follows: α-CD (3.125 g, 2.9 mmol), LiOH•H_2_O (0.235 g, 5.5 mmol), H_2_O (1.7 mL), BP (2.072 g, 10.7 mmol), H_2_O (1.9 mL). Yield: 52% (5.20 g).

BP-β-CD nanosponges were prepared similarly using the following reagent amounts: β-CD (3.674 g, 3.1 mmol), LiOH•H_2_O (0.375 g, 8.9 mmol), H_2_O (2.2 mL), BP (1.795 g, 9.25 mmol), H_2_O (1.7 mL). Yield: 47.4% (5.50 g).

### Characterization of CD nanosponges

#### Elemental analysis

Elemental analyses were performed by the Analytical Laboratory of the Polytechnic University of Milano, Italy, using a Mettler Toledo Elemental Analyzer.

#### FTIR analysis

The FTIR spectra were collected on solid samples dispersed in KBr pellets using a Perkin Elmer 100 spectrometer, as the average of 16 individual scans at 2 cm^−1^ resolution in the 4000–600 cm^−1^ interval and with corrections for atmospheric water and carbon dioxide.

#### Swelling tests

Dry finely ground samples (30 mg) were suspended in ethanol (7 mL) and allowed to settle. The supernatant liquid was then carefully removed and the solid suspended three times in either water or 0.01 M PBS pH 7.4, gently shaken for 15 min, and then allowed to settle. Finally, the swelling degree (SD) was evaluated at equilibrium using Eq. (1):

1 $$SD \left(\%\right)= \frac{{V}_{t}}{{V}_{t0}} \cdot 100$$

where *V*_*t*_ is the volume of the swollen nanosponge at equilibrium and *V*_*t*0_ is the volume of the dry sample.

#### Thermal analyses

Differential scanning calorimetry (DSC) analyses were performed with a heat flux Mettler Toledo DSC823 calorimeter on 10 mg samples sealed in standard aluminum pans under a 20 mL min^−1^ nitrogen flow and with a 20 ℃ min^−1^ heating/cooling rate. Empty pans were used as references. The analyses consisted of first heating run from 25 to 200 ℃, followed by a cooling run from 200 to 25 ℃ and second heating run from 25 to 300 ℃. Thermogravimetric (TG) analyses were performed with a Perkin Elmer TGA analyzer 4000 on 10 mg samples, in the 30–600 ℃ range, under a 50 mL min^−1^ nitrogen flow and with a 20 ℃ min^−1^ heating rate.

### Assessment of the o-toluidine sorption ability of CD nanosponges

#### o-Toluidine detection

All experiments were carried out using an Autolab PG-Stat 12 (Ecochemie, the Netherlands) potentiostat/galvanostat, according to a previously optimized method (Pifferi et al. 2014). The voltammetric cell was conical (5 mL operating volume), including an Ag|AgCl|saturated KCl electrode as a reference, a Pt wire as a counter, and a multi-walled carbon nanotubes (MWCNT)-modified glassy carbon (GC) electrode as working electrodes, respectively. The GC electrode surface was initially cleaned with synthetic diamond powder (Aldrich, diameter 1 µm) on a Struers DP Nap wet cloth. Subsequently, 20 μL of a suspension of MWCNT in dimethylformamide (0.5 mg mL^−1^) was deposited on a glassy carbon electrode and dried at 25 ℃ until complete evaporation of the casting solvent.

The working cell was thoroughly cleaned with nitric acid and ultrapure water (MilliQ^®^ Millipore) before each experiment. The sorption kinetics was assessed by monitoring the corresponding voltammetric peak currents during linear sweep voltammetry (LSV) experiments. To each sample of sorption solution (0.6 mL), 0.6 mL of 0.2 M HCl (ultrapure Fluka) was added as a supporting electrolyte before measurement. Voltammograms were recorded from the lowest concentration to the highest one. Peak heights were obtained after background subtraction.

#### Assessment of o-toluidine sorption ability

The ability to sorb *o*-toluidine from the CD nanosponges and the parent soluble α-, β-, and γ-CDs was determined by monitoring *o*-toluidine depletion from an aqueous solution of 10^−4^ M (10 ppm) in batch reactors under slight magnetic stirring. The selected starting concentration of *o*-toluidine was one order of magnitude higher than the UK maximum exposure limit (Gregg et al. 1998).

As regards α-, β-, and γ-CDs, the sorbent (30 mg) was dissolved in 100 mL 10^−4^ M *o*-toluidine solution. As regards nanosponges, the sorbent (30 mg) was put in a small punctured paper bag (tea bag) and subsequently inserted in 100 mL 10^−4^ M *o*-toluidine solution. This procedure allowed a facile sorbent separation from the decontaminated water at the end of the experiment.

#### Nanosponge regeneration after o-toluidine sorption

The *o*-toluidine-loaded samples (30 mg) were soaked in 70:30 methanol/water (20 mL) and shaken for 20 min. The sorbent was then recovered, thoroughly rinsed with water to completely remove methanol, and finally re-used for *o*-toluidine sorption. The sorption–desorption cycles were repeated twice.

## Results and discussion

### Synthesis of CD nanosponges

**Scheme 1:**
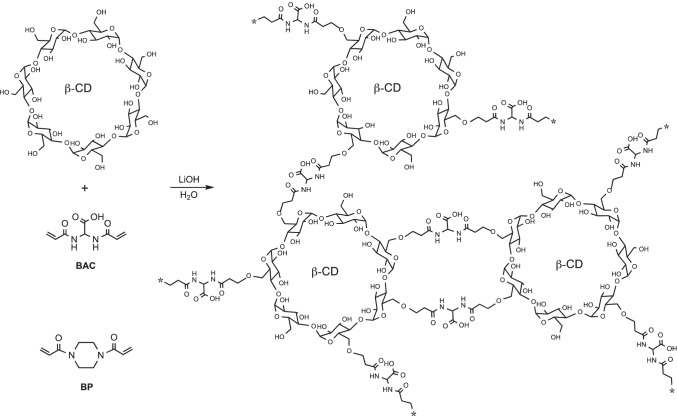
Synthesis of BAC-CD and BP-CD nanosponges. As an example, the structure of the reaction product of β**-**CD with BAC is shown in detail

The synthetic procedure adopted for preparing CD nanosponges involved the activation of the CD hydroxyl groups by deprotonation in water at pH ≥12 (Scheme 1). Under these conditions, α- and β-CD’s behaved as multifunctional monomers giving crosslinked resins (i.e., nanosponges) by one-pot polyaddition with bisacrylamides. No additional solvents or catalysts were added. A neutral bisacrylamide (1,4-bisacryloylpiperazine, BP) and a strongly acidic carboxylated bisacrylamide (2,2-bisacrylamidoacetic acid, BAC) were employed. The latter was chosen to ascertain whether its acidic nature could favor the sorption of *o*-toluidine by establishing ionic interaction. With γ-CD, the same reaction succeeded only in the case of BAC, while BP failed to produce insoluble resins. Therefore, BAC-γ-CD nanosponge was the only γ-CD-based one prepared.

For the sake of simplicity, Scheme 1 assumes that the polyaddition involved only the primary CD hydroxyl groups, neglecting the secondary ones. This, however, was not proven, and no reasonable predictions in this regard can be made. In fact, whereas the primary hydroxyl groups are less sterically hindered, the secondary ones are stronger acids, hence preferentially ionized (Loftsson and Brewster 1996). The bisacrylamide/CD molar ratio in the feed (Table 1) was adjusted to prepare nanosponges with maximum CD content, hence, presumably, maximum sorption capacities. As stated above, only BAC gave nanosponges with all three CDs, while BP failed in giving nanosponges with γ-CD. All nanosponges were purified by repeatedly soaking with water, then with ethanol, and finally dried to constant weight at 50 ℃ and 0.2 tor. Their compositions, as determined by elemental analysis, are reported in Table 1. On the whole, the CD content of the resultant resins ranged between 0.60 mmol CD g^−1^ and 0.69 mmol CD g^−1^ nanosponge (67.0 wt% and 78.0 wt%) and, for all nanosponges, was higher than in the reactant mixtures, probably due to the loss of residual unreacted bisacrylamides or soluble CD/acrylamide oligomers. This loss was apparently slightly more pronounced for α-CD-based nanosponges. The bisacrylamide moiety/CD molar ratio, calculated from elemental analyses data, ranged between 1.80 and 2.54. The lowest ratio was observed for BAC-γ-CD, the only γ-CD resins obtained, while in all other cases, it was greater than 2.21. This result is in line with the higher water sorption of BAC- γ-CD (see below), due to a lower crosslink extent of this nanosponge.

**Table 1:** Elemental analyses and CD contents of BAC-CD and BP-CD nanosponges

Sample	C%		N %			Bisacrylamide/CD (mmol mmol^−1^)		CD (mmol g^−1^ nanosponge)
	Feed^a^	Found^b^	Feed^a^	Found^b^	Feed^a^	Found^b^	Feed^a^	Found^b^
BAC-α-CD	46.17	42.42	6.03	4.82	3.66	2.54	0.59	0.68
BAC-β-CD	45.75	42.12	5.11	4.03	3.25	2.29	0.56	0.63
BAC-γ-CD	45.50	40.55	3.89	3.05	2.48	1.80	0.56	0.60
BP-α-CD	51.82	44.29	6.12	4.72	3.69	2.44	0.59	0.69
BP-β-CD	50.32	44.13	4.88	3.96	2.56	2.21	0.61	0.64

^a^Feed: expected according to the preparation recipe

^b^Found: as determined from elemental analyses data

**Fig. 1:**
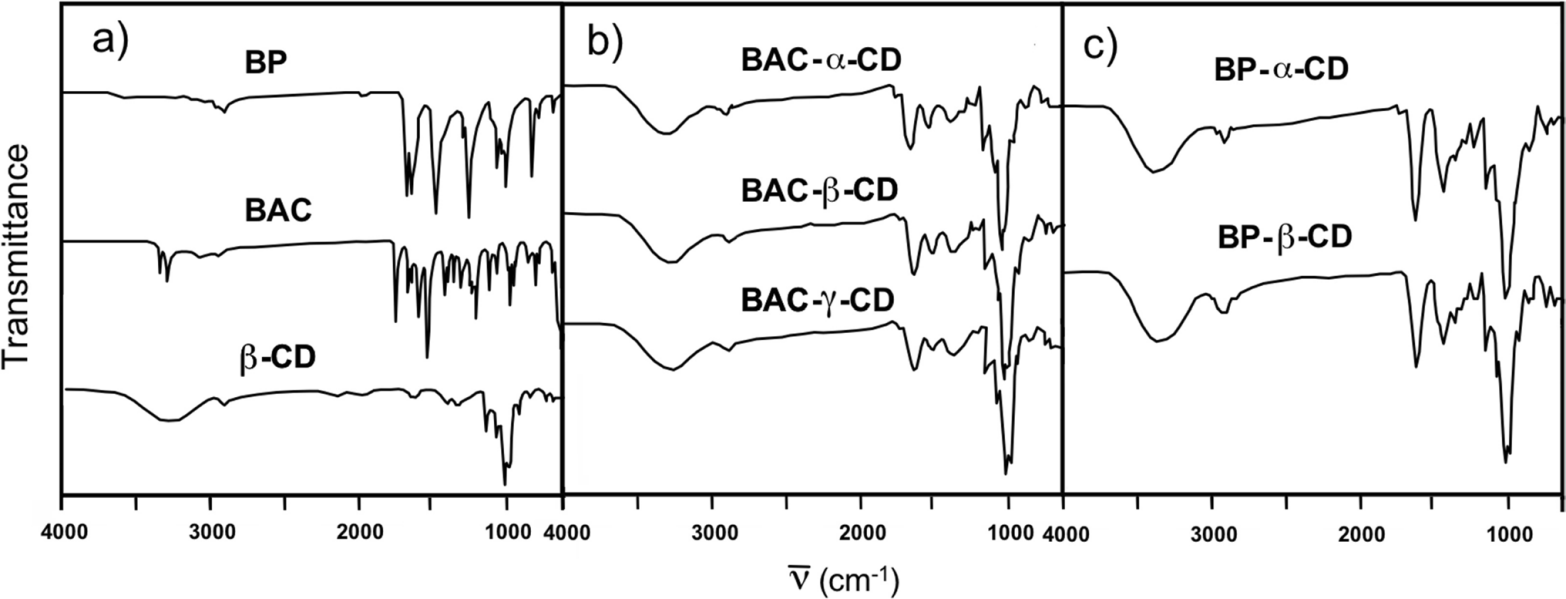
FTIR spectra of (**a**) native BP, BAC, and β-CD; (**b**) BAC-CD nanosponges; (**c**) BP-CD nanosponges

All nanosponges were characterized by FTIR spectroscopy. The spectra (Fig. 1) showed intense diagnostic bands typical of parent CDs and their addition products with either BAC or BP.

In particular, the characteristic -OH, -CH_2_, and C-O stretching bands of the CD patterns were observed at 3283 cm^−1^, 2924 cm^−1^, and 1024 cm^−1^, respectively. Meanwhile, in the BAC-based nanosponges, the diagnostic amide C=O stretching and NH bending bands of BAC addition residues were observed at 1616 cm^−1^ and 1515 cm^−1^, respectively. The same bands for BP addition residues, with the exception of the NH bending bands of BAC, were observed at 1616 cm^−1^ and 1436 cm^−1^. The -CH_2_- groups, present in all amide-deriving structural units, gave rise to the C-H stretching and bending bands observed at 2924 cm^−1^ and 1380 cm^−1^, respectively. The broad band typical of the N-H amide stretching, expected at 3283 cm^−1^ for BAC-deriving nanosponges, overlapped with the OH stretching band of CD.

**Fig. 2:**
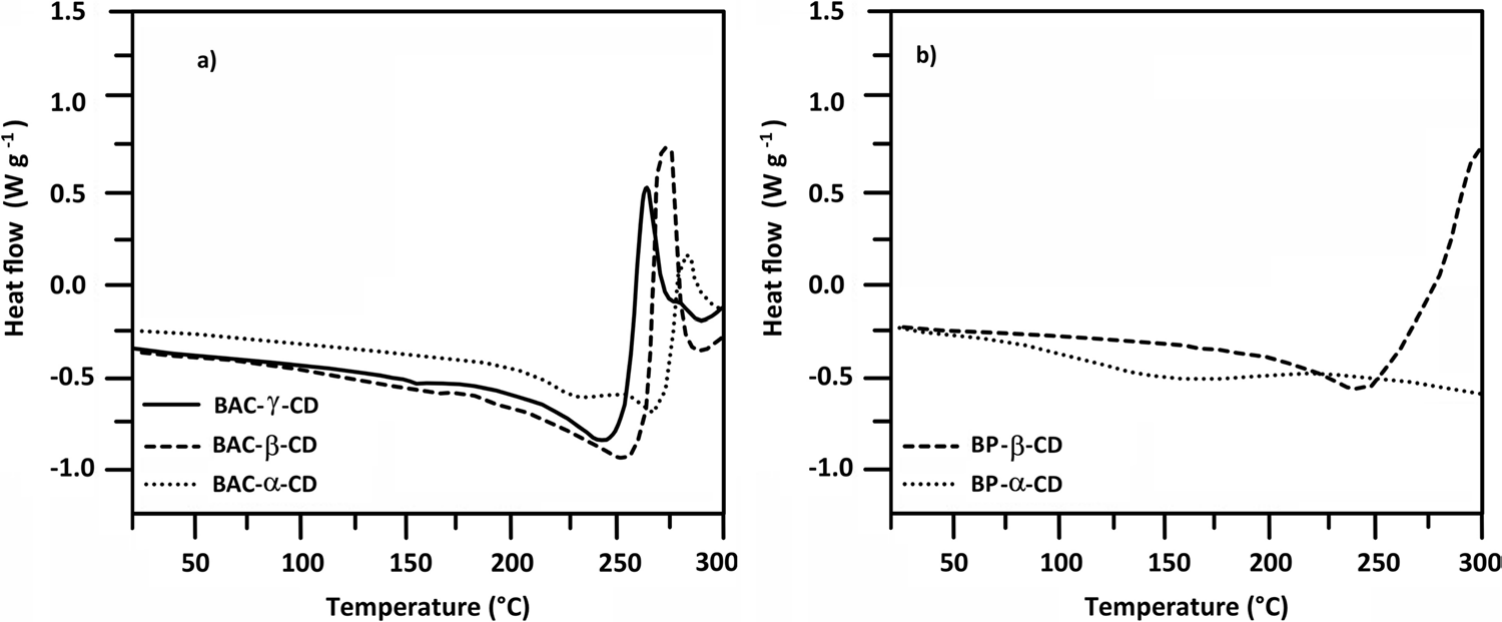
DSC thermograms of BAC-CD and BP-CD nanosponges. Heating rate: 20 ℃ min^**−**^.^1^

All nanosponges were characterized by differential scanning calorimetry (DSC) and thermogravimetric analysis (TGA). The DSC traces of the second heating cycle following a cooling cycle carried out with the same rate (20 ℃ min^−1^) (Fig. 2) showed substantially flat curves in the 20–150 ℃ range, with no evidence of transition temperatures. The thermal decomposition onset was observed at temperatures higher than 200 ℃, in line with previously published results obtained with differently crosslinked CD-based resins (Krause et al. 2010).

**Fig. 3:**
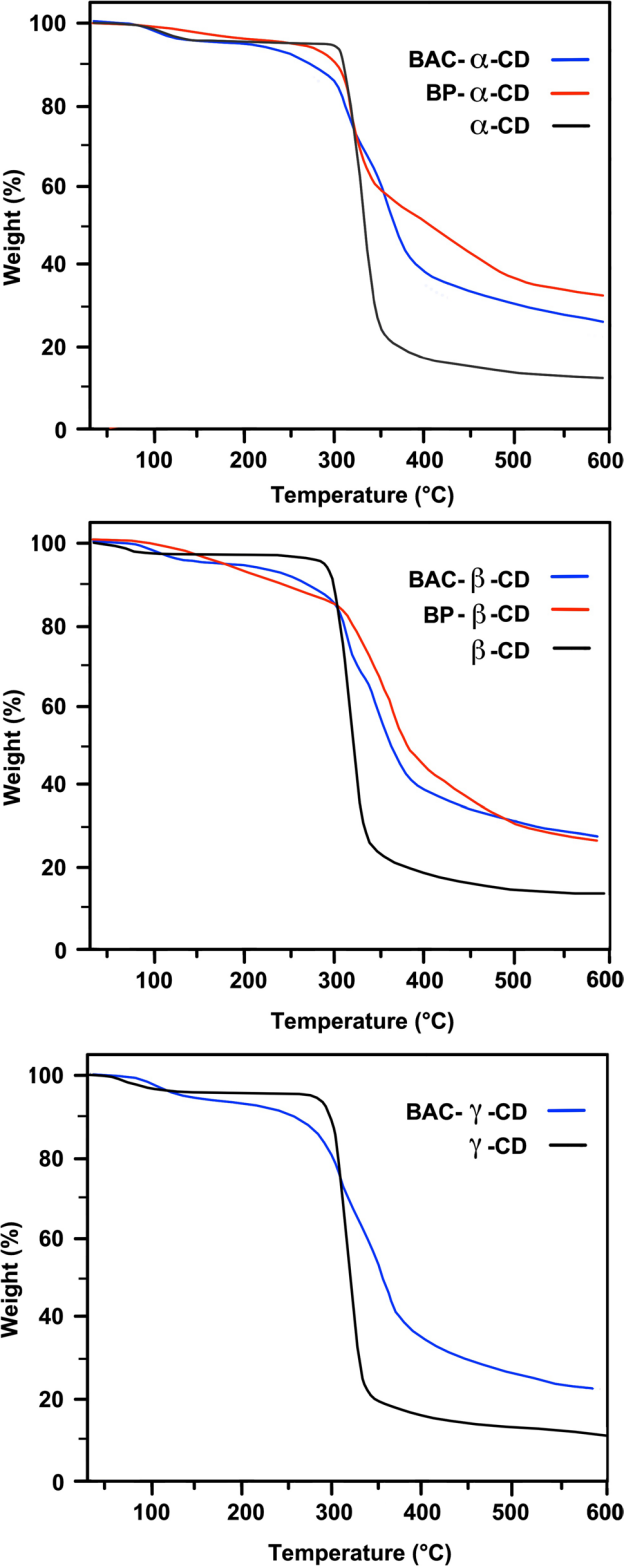
TG traces of BAC-CD and BP-CD nanosponges obtained in nitrogen. Heating rate: 20 ℃ min^**−**^.^1^

The thermal stability of BAC-CD and BP-CD nanosponges was compared with that of parent CDs, as shown by the TG traces displayed in Fig. 3 and by the collected data reported in Table 2. In line with previous observations on the thermal behavior of CD nanosponges obtained using different crosslinkers (Kumar et al. 2018; Salgin et al. 2017), the decomposition patterns of BAC-CD and BP-CD appeared to be far more complex than those of plain cyclodextrins.

**Table 2:** Thermal data collected by TG analyses in nitrogen

Sample	T_onset10%_^a^ (℃)	T_max1_^b^ (℃)	T_max2_^b^ (℃)	RMF_600_^d^ (%)
α-CD	306	333	-	11.5
β-CD	311	333	-	12.5
γ-CD	303	332	-	11.0
BAC-α-CD	292	296	348	30.0
BAC-β-CD	290	287	345	31.0
BAC-γ-CD	285	285	345	23.0
BP-α-CD	302	298	445	33.0
BP-β-CD	252	-	342	30.5

^a^Onset decomposition temperature at 10% weight loss

^b^Temperature at maximum weight loss rate for the 1st decomposition step

^c^Temperature at maximum weight loss rate for the 2nd decomposition step

^d^Residual mass fraction at 600 ℃

In agreement with literature reports (Trotta et al. 2000), the TG curves of cyclodextrins had a similar three-stage profile, including (i) a first stage of dehydration, up to 120 ℃, in which sorbed and crystallization water was removed; (ii) a second stage, from 250 to 350 ℃, associated with a T_onset10%_ around 300 ℃, a maximum weight loss of 75–80% at T_max1_ 332–333 ℃, and the formation of a thermally stable residue, called char; and (iii) a third stage at temperatures above 400 ℃ in which char formed in the previous stage progressively degraded down to a residual mass fraction at 600 ℃, RMF_600_, of 11.0–12.5%. The main decomposition phase centered at T_max1_ was attributed to the opening of the cyclodextrin ring, followed by structural transformations similar to those of cellulose, which include the loss of glycosidic and hydroxyl groups and the formation of double bonds, carbonyl groups, and aromatic structures.

The TG patterns of BAC-CD and BP-CD nanosponges exhibited T_onset10%_ values invariably lower (Table 2) than those of cyclodextrins. Moreover, the weight loss patterns proceeded through four decomposition steps (Fig. 3) down to a significantly higher RMF_600_ of 23.0–33.0%. In detail, apart from the dehydration stage, up to 120 ℃, a second weight loss of approximately 5–15% occurred in the 150–300 ℃ range, ascribed to the cleavage of the C–C bond between CD and the linker. The third stage of degradation, ascribed to CD decomposition, was found at slightly different temperatures depending on the nature of the linker. All BAC-CD nanosponges had similar T_max2_, around 345 ℃, with a weight loss of around 45% in the range of 300–400 ℃. Regarding BP-CD nanosponges, BP-α-CD exhibited a T_max2_ value of 445 ℃ and BP-β-CD of 342 ℃. The fourth step (400–600 ℃ and 500–600 ℃ for BAC- and BP-based cyclodextrins, respectively) was ascribed to the relatively slow thermal degradation of the char formed in the previous step.

**Fig. 4:**
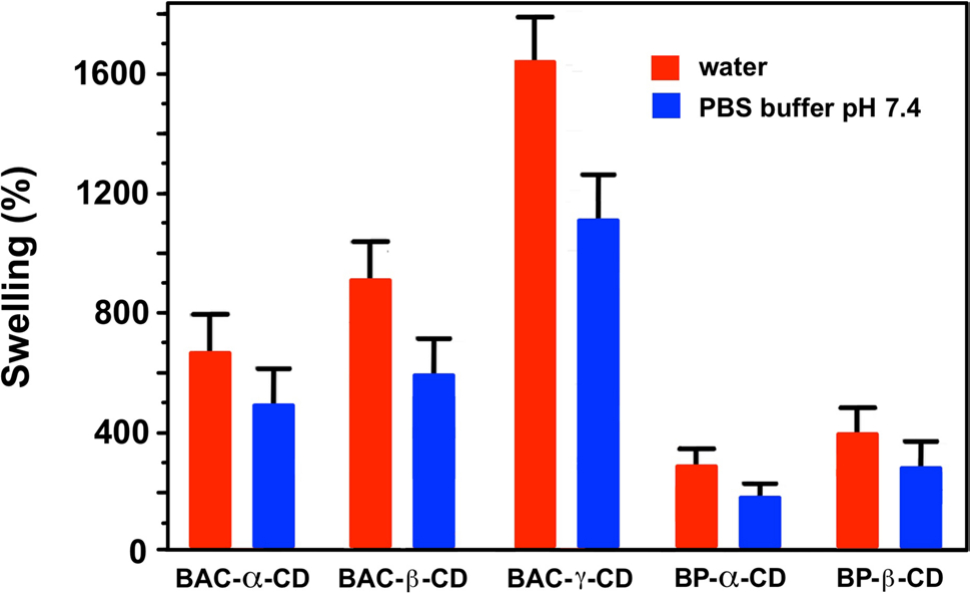
Swelling behavior of BAC-CD and BP-CD nanosponges in water and PBS buffer pH 7.4

The swelling ability of nanosponges was determined both in water and 0.01 M PBS pH 7.4. The results are shown in Fig. 4. The swelling in water was higher than in buffer and, for nanosponges with the same crosslinking arm, increased in the order α-CD < β-CD < γ-CD. The swelling degree is also influenced by the structure of the crosslinking arm. Under the same conditions, BAC nanosponges constantly swelled more than those based on the same CD, but deriving from BP, possibly due to the ionic nature of BAC.

### o-Toluidine sorption

The o-toluidine sorption ability of CD nanosponges was assessed by monitoring the o-toluidine depletion from 10^−4^ M (about 10 ppm) aqueous solutions in linear sweep voltammetry experiments, making use of MWCNT-modified glassy carbon (GC) electrodes, a robust and sensitive analytical method that has proven to detect o-toluidine down to 0.16 ppm with an apparent recovery factor of about 100% (Pifferi et al. 2014). The sorption study was made at the natural pH in which o-toluidine is present in water-polluted samples. Moreover, the range of pollutant concentration investigated in this work was really low, thus not influencing the solution pH during the sorption.

**Fig. 5:**
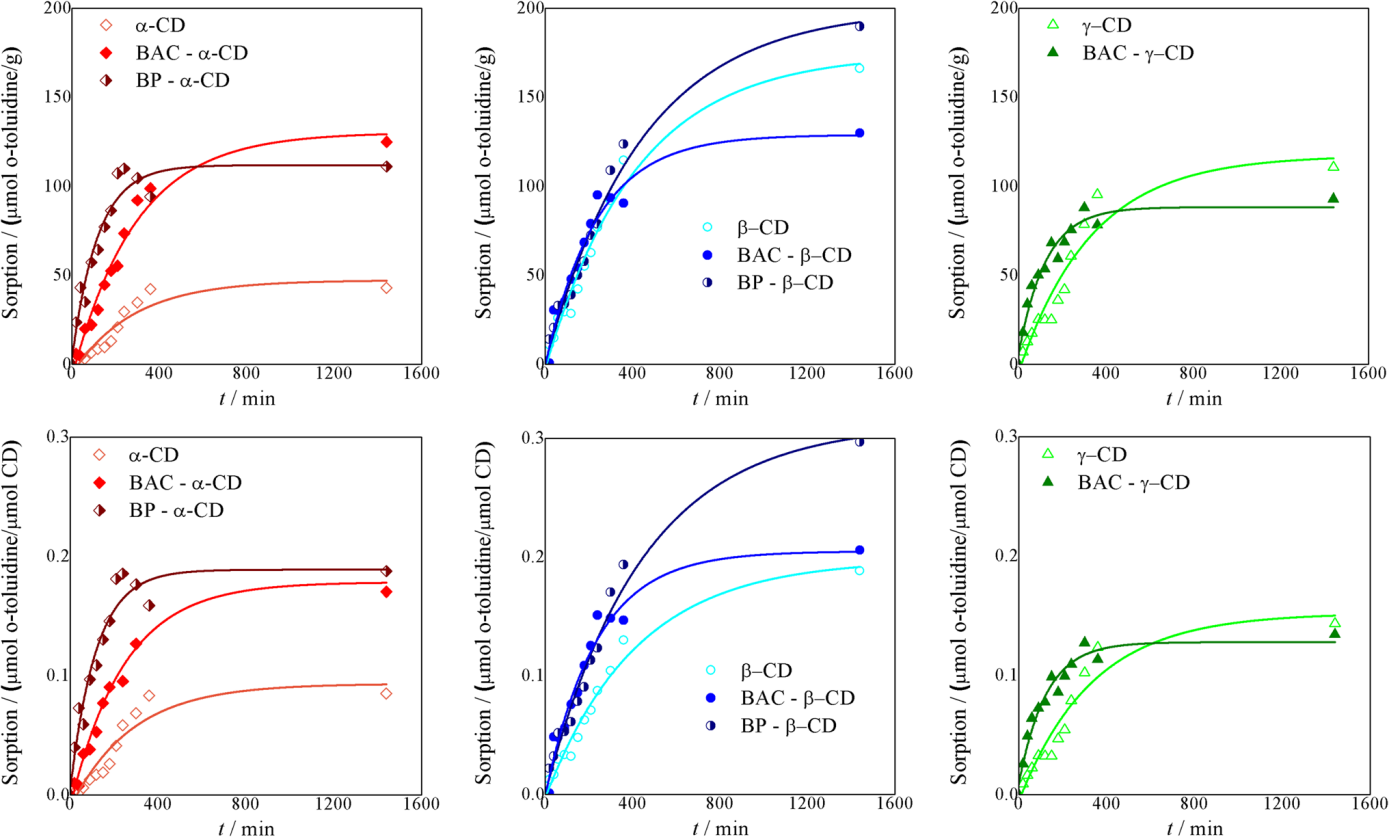
*o*-Toluidine sorption by soluble CDs, and CD-containing nanosponges referred to the CD (**a**) mass unit and (**b**) molar content in the sorbent [sorption experimental conditions: o-toluidine from an aqueous solution of 10^**−**4^ M (10 ppm) in batch reactors under slight magnetic stirring]; corresponding mono-exponential kinetics in full lines

The observed sorption capacities were compared with those of native soluble α-, β-, and γ-CDs. In all cases, the sorption kinetics, expressed both referring to the sorbent mass unit (Fig. 5a) and the molar CD content of the sorbent (Fig. 5b), were found to fit a mono-exponential kinetic model derived from a more conventional double-exponential model in which the second term prevails over the second one, indicating a good homogeneity of the sorbent materials (Ferruti et al. 2006), and the absence of adsorptive phenomena in competition with sorption.

The mono-exponential model implies the following equations (Eqs. (2) and (3)):

2 $${q}_{t}={q}_{e}-{\frac{D}{{m}_{\mathrm{sorb}}}e}^{-{k}_{D}t}$$

3 $$q_t\prime=q_e\prime-{\frac{D^\prime}{{\mu mol}_{CD}}e}^{-k_Dt}$$

where *m*_sorb_ is the weight of the sample of the sorbing material, μmol_CD_ is the quantity of CD present in the sorbing material, *k*_D_ is the relevant sorption rate constant, *q*_e_ and *q*_e_’ are the equilibrium *o*-toluidine sorption capacities, and *D* and *D*’ are further parameters modulating the sorption rate. The relevant kinetic parameters obtained for all tested samples are reported in Table 3.

**Table 3:** Experimental *q*_e_ values and kinetic parameters for the sorptions of *o*-toluidine from 10^−4^ M (10 ppm) aqueous solutions by CD-based nanosponges [sorption experimental conditions: o-toluidine from an aqueous solution of 10^−4^ M (10 ppm) in batch reactors under slight magnetic stirring]

Sample	Sorption performance per g of sorbent			Sorption performance per µmol of CD in the sorbent	
	*q*_e_ (μmol g^−1^)	*D* (μmol L^−1^)	*q*_e_’ (μmol μmol^−1^)	*D’* × 10^−3^ (μmol L^−1^)	*k*_D_ × 10^−3^ (min^−1^)
α-CD	47 ± 6	1.6 ± 0.2	0.05 ± 0.01	0.0031 ± 0.0004	3.6 ± 0.9
BAC-α-CD	104 ± 7	3.5 ± 0.2	0.15 ± 0.01	0.0038 ± 0.0002	4.1 ± 0.6
BP-α-CD	112 ± 7	3.0 ± 0.2	0.16 ± 0.01	0.0037 ± 0.0003	8 ± 2
β-CD	174 ± 9	5.3 ± 0.3	0.20 ± 0.01	0.0060 ± 0.0003	2.4 ± 0.3
BAC-β-CD	137 ± 5	4.2 ± 0.2	0.24 ± 0.01	0.0042 ± 0.0002	3.4 ± 0.3
BP-β-CD	199 ± 10	6.3 ± 0.3	0.29 ± 0.01	0.0062 ± 0.0003	2.3 ± 0.2
γ-CD	117 ± 11	3.6 ± 0.3	0.15 ± 0.01	0.0047 ± 0.0005	3.0 ± 0.6
BAC-γ-CD	88 ± 4	2.7 ± 0.2	0.14 ± 0.01	0.0024 ± 0.0002	8 ± 1

The sorption efficiency (see *q*_e_ and *D* values) of β-CD nanosponges is higher than that of α-CD- and γ-CD- nanosponges in line with the higher efficiency of β-CD compared with that of both α-CD and γ-CD, due to the specific capacity of the internal cavity size of β-CD to create stable inclusion complexes with monocyclic aromatic compounds (Szejtli 1998).

The equilibrium sorption capacities of both α-CD and β-CD nanosponges, expressed as mol *o*-toluidine mol^−1^ CD (*q*_e_’ factors, accounting for the number of sorption sites), were remarkably higher than those of parent cyclodextrins, particularly for α-CD nanosponges. This was ascribed to two concurrent factors: the cooperation of correctly oriented cyclodextrin groups in forming inclusion complexes with *o*-toluidine and the additional uptake of *o*-toluidine by the interstitial channels created by the interconnected arms. On the opposite, the sorption capacity of BAC-γ-CD was approximately the same as that of γ-CD. This could be attributed to the fact that due to the larger size of the internal cavity size of γ-CD and to the lower crosslinking degree that probably leads to wider interstitial channels in γ-CD nanosponges, *o*-toluidine complexation does not benefit from the establishment of cooperative effects between adjacent CD macrocycles.

It may also be observed that the BP-based nanosponges performed better than their BAC counterparts, more significantly in the case of BP-β-CD. This behavior was ascribed to the higher affinity of the BP-deriving interconnecting arms for *o*-toluidine. The above-reported assumption that owing to its acidic nature, the BAC interconnecting arms could favor the sorption of *o*-toluidine by establishing ionic interactions did not materialize.

As regards *o*-toluidine removal from water, it is worth mentioning that the best results obtained in this work are significantly superior to those of other *o*-toluidine sorbents. In particular, the *o*-toluidine abatement dropped to 0.16 ppm, which represents the detection limit of the adopted analytical technique, lower by one order of magnitude than the figures reported so far for all other sorbents (Sun et al. 2010). In addition, all nanosponges, irrespective of the size of the CD inner cavity, proved capable of removing *o*-toluidine from dilute aqueous solutions under conditions similar to those usually found on the field in most *o*-toluidine-polluted aquifers, abating it down to at least 0.16 ppm. Remarkably, the fact that all three CDs lead to effective nanosponges allows us to conclude that high-performance *o*-toluidine sorbents can be prepared from commercially available crude CD blends, which are very cheap compared to pure samples of α, β, and γ-CD used in this pioneering work. This is of paramount importance in view of the mass production of CD-bisacrylamide nanosponges for water purification.

### Nanosponge regeneration

**Fig. 6:**
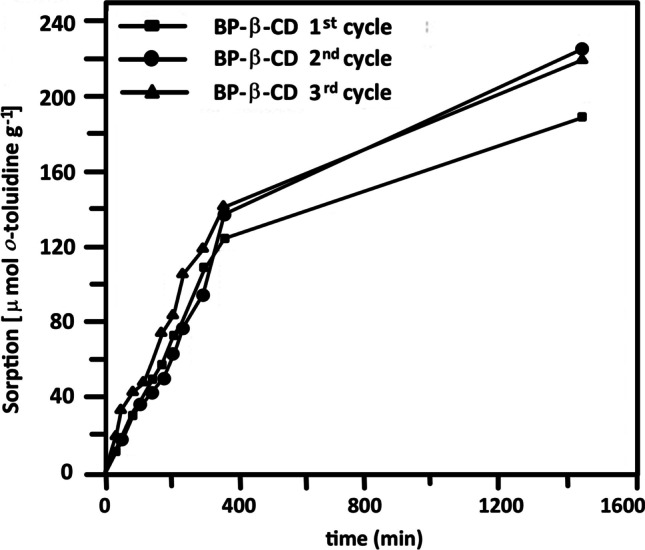
Sorption capacities of *o*-toluidine for BP-β-CD nanosponges before (1st cycle) and after recycling (2nd and 3rd cycles)

Regeneration studies were performed for BP-β-CD, the sorbent with the highest *o*-toluidine equilibrium sorption. The nanosponge was simply regenerated by methanol extraction of the sorbed material. Three *o*-toluidine sorption-desorption cycles were performed. The sorption capacity after regeneration was monitored each time and compared with that of the pristine nanosponge. Interestingly, the sorption capacity (Fig. 6) slightly improved after the first regeneration treatment, possibly due to a rearrangement of the nanosponges network that facilitated the *o*-toluidine inclusion in a sort of imprinting of the polymer. More investigations should be done to prove this preliminary idea.

## Conclusions

Tailor-made α-, β-, and γ-CD-based nanosponges characterized by high CD content, in which the CD moieties were covalently connected by either ionizable or neutral bisacrylamide-deriving arms, were synthesized from cyclodextrins and bisacrylamides by base-catalyzed stepwise oxo-Michael polyaddition in aqueous solution. These nanosponges exhibited high water sorption ability and were thermally stable at least up to 250 ℃. Their TG patterns significantly differed from those of the parent CD and were in line with those of previously reported CD nanosponges. These CD nanosponges removed *o*-toluidine from 10^−4^ M (~10 ppm) aqueous solutions (the reference UK-approved maximum exposure limit) down to the detection limit of the adopted analytical technique (0.16 ppm).

The nanosponges’ sorption capacities were in the range of 88–199 µmol g^−1^ (10–21.3 mg g^−1^), one order of magnitude higher with respect to other CD-containing sorbents reported in the literature (Sun et al. 2010). Interestingly, the sorption capacity of nanosponges toward *o*-toluidine was even higher than that of the corresponding cyclodextrins. This can be attributed to the participation in the sorption process of the interstitial channels between the CD units.

The sorbents were simply regenerated by extracting with methanol and recycled with no loss of performance. On the opposite, their sorption capacity slightly increased, possibly due to some molecular imprinting during the first sorption/desorption cycle. Also considering the simplicity, potential scalability (the preparation process can be extended to any compound containing two activated acrylamide double bonds (Ferruti et al. 2019)), environmental sustainability of their preparation, and no phyto-toxicity (Alongi et al. 2022), these cyclodextrin-based materials warrant potential as *o*-toluidine sorbents competitive with most materials presented so far to the same purpose and deserve further investigations aimed at widening their scope as sorbents of organic water pollutants other than *o*-toluidine.

## Data Availability

The main data generated within this research are included in the paper. Some further data that support the findings are available from the corresponding author upon reasonable request.
